# How Does Physical Activity During Youth Affect the Development of Multisite Musculoskeletal Pain or Discomfort in Young Adults?

**DOI:** 10.1155/prm/8810256

**Published:** 2025-11-29

**Authors:** Emilie Aarestrup Larsen, Johan Hviid Andersen, David Høyrup Christiansen, Trine Nøhr Winding

**Affiliations:** ^1^Department of Occupational and Environmental Medicine, Goedstrup Hospital, Herning, Denmark; ^2^Department of Medicine, Viborg Regional Hospital, Viborg, Denmark; ^3^Department of Clinical Medicine, Aarhus University, Aarhus, Denmark; ^4^Elective Surgery Centre Silkeborg Regional Hospital, University Clinic for Interdisciplinary Orthopaedic Pathways, Silkeborg, Denmark; ^5^Center for Research in Health and Nursing, Viborg Regional Hospital, Viborg, Denmark

## Abstract

**Background:**

Multisite musculoskeletal pain is a common condition among young adults, and identifying risk factors for the experience of pain is of particular interest. During the transition from adolescence to adulthood, individuals become less physically active. The aim of this study was to investigate the long-term association between physical inactivity during adolescence and young adulthood and multisite musculoskeletal pain or discomfort later in young adulthood, with analyses conducted separately for males and females.

**Methods:**

Data on self-reported levels of physical activity at Ages 15, 18, and 21 and multisite musculoskeletal pain or discomfort at Age 28 from The West Jutland Cohort Study (*n* = 1833) were used. The levels of physical activity were dichotomized into low level (< 4 h per week) and high level (> 4 h per week) of physical activity. Logistic regression analysis was conducted, stratified by sex and adjusted for depressive symptoms, smoking, parental and own educational level, equivalized childhood income, and pain status at Age 15. Results were presented as odds ratios with corresponding 95% confidence intervals.

**Results:**

The prevalence of multisite musculoskeletal pain or discomfort was 32%, and more females reported experiencing multisite pain (37%) than males (26%). The adjusted results showed that adolescents and young adults who were physically inactive were more likely to experience multisite musculoskeletal pain or discomfort later in life than those adolescents and young adults who were physically active (OR 1.9 (1.3–2.9)).

**Conclusion:**

Physical inactivity during adolescence and young adulthood is a risk factor for experiencing multisite musculoskeletal pain or discomfort at Age 28. This emphasizes the importance of physical activity during adolescence and young adulthood for later physical health.

## 1. Introduction

Musculoskeletal pain is a prevalent and recurrent condition, often manifesting in multiple anatomical regions simultaneously [1]. Studies show that up to 40% of adolescents experience multisite musculoskeletal pain over a six-month period [2–4]. This condition appears to persist over time, as children and adolescents with multisite musculoskeletal pain often continue to experience pain into adulthood [5–7].

In recent years, several potential risk factors for both single-site and multisite musculoskeletal pain have been identified, including smoking [8–10], depressive symptoms, stress, anxiety [7, 9, 11–13], pre-existing musculoskeletal pain [7], and low socioeconomic status [9]. However, the role of sex in musculoskeletal pain remains debated. Hanvold et al. found that females report more pain and pain sites than males [5], while a systematic review by Huguet et al. found that evidence on sex as a risk factor for multisite pain is inconsistent [9].

A less studied risk factor for multisite musculoskeletal pain is physical activity. Despite the health benefits associated with regular physical activity, more than three-quarters of adolescents fail to meet the recommendations of at least 1 h of physical activity per day [14, 15]. Moreover, physical activity levels drop significantly as adolescents transition into adulthood [16, 17].

Physical inactivity may further contribute to our understanding of multisite musculoskeletal pain. Physical inactivity leads to reduced muscle strength and endurance and decreased nutritional supply to skeletal joints, thereby accelerating joint degeneration [18, 19]. Furthermore, it is suggested that physical activity may be protective against multisite musculoskeletal pain due to improved motor control, which facilitates adaptations that prevent stress and injury [11, 20]. Additionally, Guddal et al. note that psychosocial factors may increase the risk of physical inactivity, particularly due to lower levels of motivation [21].

Previous studies have investigated the association between physical activity and single-site [21–23] and multisite pain [2, 18, 19, 24, 25]. However, inconsistent findings, short follow-up periods, and cross-sectional designs challenge the interpretation. Only two long-term studies have examined the association between physical activity in youth and multisite musculoskeletal pain in adulthood [5, 10]. Puroila et al. found that physical inactivity was a long-term risk factor for pain in two sites among females, but not for three sites [10]. They measured physical activity only once and did not assess baseline pain status.

Hanvold et al. showed that increased physical activity during youth was associated with fewer pain sites, but the results were not statistically significant [5]. Their study population was small, and many participants were lost-to-follow-up.

Therefore, the long-term association between physical activity measured during adolescence and young adulthood and multisite musculoskeletal pain in adulthood remains unclear.

Thus, the objective of this study is to investigate how the level of physical activity during adolescence and young adulthood affects the experience of multisite musculoskeletal pain or discomfort in later young adulthood, with analyses conducted separately for males and females.

## 2. Methods

### 2.1. The Study Population

The study population was derived from the West Jutland Cohort study [26]. All individuals born in 1989 and still living in the county of Ringkjoebing, Denmark, in April 2004, 3681 adolescents, were invited to participate. A total of 3054 participated, corresponding to a response rate of 83% [26].

Questionnaire surveys have been sent out to the source population approximately every 3 years since the initial survey round when they were 15 in 2004. Register information was retrieved from Statistics Denmark by using the personal identification number from the Central Person Register, and the participants were linked to parents or guardians [27].

Participants who answered questions about physical activity at least once in 2004 (Age 15), 2007 (Age 18), and/or 2010 (Age 21) and musculoskeletal pain in 2017 (Age 28) were included in this study. This resulted in a study population of 1833 participants (49%).

### 2.2. Exposure Variables

Information about level of physical activity was gathered at three time points when participants were 15, 18, and 21 years old with the question, “How many hours a week do you exercise or play sports where you become short of breath or have to sweat?” The possible answers were none, 1/2 h, 1 h, 2–3 h, 4–6 h, or 7 h or more.

The variable was dichotomized on the basis of the recommendations for weekly physical activity by WHO [14], as a dichotomous variable with “low level of physical activity” defined as < 4 h per week and “high level of physical activity” defined as ≥ 4 h per week.

Additionally, to describe the accumulation of physical activity, participants were classified into 3 groups—“active maintainers,” “inactive maintainers,” or “fluctuaters”—based on whether they consistently maintained a low, high, or fluctuating level of physical activity determined by their responses at Ages 15, 18, and 21.

### 2.3. Outcome Variable

Multisite musculoskeletal pain or discomfort was assessed after 13 years of follow-up in 2017, when the participants were 28 years old. The following 4 items were used to create the outcome variable: “How much have you experienced pain or discomfort in: (1) neck/shoulder; (2) elbow/forearm/hands; (3) hips/knees/ankles; and (4) low back, within the last 12 months?” The response categories were none, very little, a little bit, some, a great deal, much, and extremely much. Each item was dichotomized into pain (those answering some, a great deal, much, or extremely much) or no pain (those answering none, very little, or a little bit). Multisite musculoskeletal pain or discomfort was defined as pain or discomfort in two or more locations; therefore, the outcome variable was dichotomized into no pain (0–1 site) and multisite pain (2–4 sites).

### 2.4. Covariates

The covariates depressive symptoms, smoking status, socioeconomic status, and pain at baseline were chosen as possible confounders based on a review of the literature [8–11, 13, 18].

Depressive symptoms were measured when the participants were 15, 18, and 21 years old using an abbreviated 4-item version of the Center for Epidemiological Studies Depression Scale for Children (CES-DC) [28], which measures the level of current depressive symptoms. The response categories were “not at all,” “a little,” “some” or “quite a lot,” with scores ranging between 0 and 3 points. Based on the 4 items, a total sum score was constructed with a maximum of 12 points. The final depression variable was created as the average of the sum scores from the three rounds, with participants required to have answered at least two out of the three rounds. High values corresponded to having depressive symptoms.

Smoking status was assessed when participants were 18 and 21 years old with the question “Do you smoke?” Based on the answers, the variable was dichotomized into *smoker* (yes daily or at least once a week) or *non-smoker* (never, no but formerly, or yes but rarer than once a week). If participants were categorized as smokers in at least one of the two rounds, they were classified as smokers in this study.

Pain status at baseline (age 15) was based on the following question from the Hopkins scale regarding psychosomatic symptoms [29]. “In the past weeks, to what extent have you been bothered with low back pain or/and muscle pain?” The variable was dichotomized into *absent* (not at all, a bit) or *present pain* (some, a great deal, very much).

The following three variables, based on data from Statistics Denmark [27], were used as measures of socioeconomic status: equivalized household income when participants were 7–10 years old, parents' highest educational level when the participants were 14 years old, and participants' own educational level at Age 28. This classification of socioeconomic status is consistent with previous studies on the same cohort [30–32].

The equivalized household income is a weighted measure where the first adult weights 1, individuals over 14 years of age weigh 0.5, and children weigh 0.3. The total household income is divided by the sum of the weightings. The overall household income was calculated as an average if data for at least 3 out of 4 years were available.

The parents' and participants' highest educational levels were categorized into four groups based on the length of education: < 10 years corresponding to primary school, 10–12 years corresponding to high school, technical school, or vocational school, 13–15 years corresponding to a short or medium further education (e.g., a bachelor's), or > 15 years corresponding to a long further education (e.g., a master's or PhD).

Information about sex came from registers [27].

### 2.5. Statistical Methods

Population characteristics were calculated and presented for outcome, exposures, and covariates as number and percentage or mean and standard deviation, in total and for females and males separately. We stratified by sex since we assumed that sex differences existed.

A correlation analysis between exposures and covariates revealed that no correlation exceeded *r* = 0.35, and all covariates could therefore be included in the same model.

Four separate logistic regression analyses were performed to estimate the associations between physical activity levels during adolescence and young adulthood and the occurrence of multisite musculoskeletal pain or discomfort in later adulthood. Three of the analyses were based on distinct populations at the ages of 15, 18, and 21, respectively, while the fourth analysis was based on responses collected across all three time points. The groups with the highest levels of physical activity serve as the reference groups. The analyses were adjusted for depressive symptoms, smoking, parental and own educational level, equivalized childhood income, and pain status at Age 15. Both crude and adjusted odds ratios (ORs) were presented with 95% confidence intervals. The results were presented for all as well as stratified by sex. Model fit was assessed using log-likelihood values.

Characteristics of lost-to-follow-up were shown to identify whether nonparticipants differed from participants based on register data from Statistics Denmark [27].

All statistical analyses were performed using STATA (Version 16).

## 3. Results

Descriptive statistics of the study population are presented in Table 1. By Age 28, 32% experienced multisite musculoskeletal pain or discomfort, with a higher occurrence reported among females (37%) compared to males (26%). Physical activity levels declined during adolescence and young adulthood for both sexes. At Age 15, 44% of participants had low levels of physical activity, which increased to 60% by Age 21. At all three time points a higher proportion of females (48%–65%) were active for less than 4 h per week compared to males (38%–53%). Additionally, 21% of participants consistently maintained low activity levels throughout adolescence and young adulthood, with a greater proportion of females (25%) classified as inactive maintainers compared to males (15%).

Table 2 shows characteristics of those lost-to-follow-up. This revealed that in our study population, 59% were female, and 7% had primary school as their highest educational level at Age 28, whereas in the source population, 48% were female, and 13% had primary school as their highest educational level.

Table 3 shows the results of the association between low physical activity levels during youth and multisite musculoskeletal pain or discomfort at Age 28.

At all time points low physical activity was statistically significantly associated with multisite musculoskeletal pain or discomfort in both crude and adjusted models in the total population. The strongest adjusted association was observed among females at Age 18, where those with a low level of physical activity were 1.5 times more likely to experience multisite musculoskeletal pain or discomfort by Age 28 compared to females with a high level of physical activity. No significant association between low physical activity levels and multisite musculoskeletal pain or discomfort was found among 18-year-old males.

Furthermore, when examining the accumulation of physical activity during adolescence and young adulthood, both the crude and adjusted analyses showed statistically significant increased odds of multisite musculoskeletal pain or discomfort among fluctuators and inactive maintainers compared to active maintainers in the total population. The crude association between fluctuators and multisite musculoskeletal pain or discomfort was OR 1.7 (1.2–2.4), and inactive maintainers and multisite musculoskeletal pain or discomfort was OR 2.3 (1.5–3.4). The associations only slightly attenuated when adjusted for the potential confounders (OR 1.4 (1.0–2.1) and OR 1.9 (1.3–2.9)). This indicates that individuals who remained inactive from Ages 15 to 21 had 1.9 times higher odds of experiencing multisite musculoskeletal pain or discomfort at Age 28 compared to those who remained active.

This pattern of increased odds of multisite musculoskeletal pain or discomfort among fluctuators and inactive maintainers, compared to active maintainers, was observed in both females and males; however, only female inactive maintainers showed a statistically significant association. Adjusted models demonstrated improved model fit relative to crude models, as reflected by higher log-likelihood values across all analyses.

## 4. Discussion

In this follow-up study, we found that physically inactive adolescents and young adults were more likely to experience multisite musculoskeletal pain or discomfort at Age 28 than physically active adolescents and young adults. Similar results have been shown in the general population [20, 33]. In a Norwegian study, Kamaleri et al. suggested that low levels of physical activity were associated with an increased number of pain sites [33].

Contradictory findings have also been reported. For example, Paananen et al. found that high levels of physical activity were associated with multisite musculoskeletal pain among 16-year-old Finns [18]. However, as this study employed a cross-sectional design, the direction of the relationship remains uncertain. Additionally, in a 2-year follow-up study, Jussila et al. found a high level of physical activity to be associated with the persistence of multisite musculoskeletal pain at Age 18 [13]. The difference between our findings and those of Jussila et al. may be explained by their shorter follow-up period of 2 years, in contrast to our 13-year follow-up assessing multisite pain at Age 28. While high physical activity during adolescence may increase short-term risk due to overuse or injury, it could have a protective effect over the long term.

In addition to physical inactivity, our findings highlight depressive symptoms, smoking, and baseline pain as significant risk factors for multisite musculoskeletal pain in adulthood. These results align with previous studies showing that psychological distress can increase pain sensitivity and chronicity [7, 11]. Smoking has also been identified as a risk factor for chronic musculoskeletal pain, potentially due to its impact on inflammation and tissue healing [8]. Moreover, the presence of previous pain is a known predictor of future pain, emphasizing the importance of early pain management [9].

The estimates changed slightly (between 0.1 and 0.4) after adjustment for the potential confounders: depressive symptoms, smoking, pain at baseline, and socioeconomic status. Although these covariates underscore the multifactorial nature of multisite pain, suggesting that interventions should address both physical and psychosocial factors, they do not show any great confounding impact on the association between physical activity in adolescents and young adults and multisite musculoskeletal pain or discomfort.

Furthermore, our results showed sex differences, with statistically significant associations between low physical activity levels at Ages 15, 18, or among inactive maintainers and multisite musculoskeletal pain or discomfort in females. In contrast, no statistically significant associations were observed among males. This is in line with the findings of Puroila et al. [10]. In a 17-year follow-up study, they found an association between low physical activity when the participants were 14 years old and multisite musculoskeletal pain at Age 31 among females, whereas the association was unclear among males [10]. The difference between the two sexes in this study could be due to a lack of power. There is a difference in significance, but the ORs are of fairly similar strength and show the same trends in terms of physical activity and later multisite pain.

A study by Kamada et al. found an association between physical activity and the experience of multisite musculoskeletal pain in individuals aged 15–18. They studied physical activity as sport participation and found that those engaging in more than 360 min of sport per week had a higher risk of multisite pain compared to those participating for less than 100 min per week [34]. This finding is consistent with findings by Guddal et al., who examined both self-reported overall physical activity and sport participation among adolescents. They found that moderate levels of physical activity were associated with a reduction in single-site pain, whereas sport participation was linked to increased odds of lower extremity and low back pain [21].

The different measurements of physical activity make it difficult to compare with our study and may possibly explain the different findings.

Sports participation and general physical activity, while overlapping especially in younger adolescents, differ in important ways. Sports often involve structured, repetitive, and higher-impact movements, which can increase the risk of localized pain and injuries [21]. In contrast, general physical activity is typically more varied and may be less likely to cause specific tissue damage. This distinction could explain why sports participation and overall physical activity sometimes show different associations with pain later in life.

When considering multisite musculoskeletal pain, sport participation may play a less significant role, as multisite pain can be seen as a more generalized form of pain, potentially linked to increased pain sensitivity [35]. Pain is a subjective experience best understood within a biopsychosocial framework [35], a perspective further supported by the identification of psychosocial factors as key risk factors for multisite pain [7, 9, 11, 13, 18].

The influence of sport participation on multisite musculoskeletal pain was not examined in the present study. Consequently, it remains uncertain whether the inclusion of such data would have yielded a more nuanced understanding of the relationship between physical activity and multisite pain.

### 4.1. Methodological Considerations

A strength of this study is the prospective design with 13 years of follow-up, which enabled us to examine the long-term association between physical activity and multisite musculoskeletal pain or discomfort. However, the long follow-up period may also be a limitation, as changes in physical activity patterns over time could affect the accuracy of exposure classification and potentially dilute the observed association.

The measurement of physical activity at several time points during adolescence and young adulthood allowed us to identify patterns in physical activity levels. Collecting data at several time points reduces the risk of self-reported information being subject for inaccuracies and recall bias, but still there is a risk of introducing misclassification when dichotomizing the exposure variable and relying on self-reported information. If physically inactive individuals tend to overestimate their activity levels, while physically active individuals report more accurately, this could introduce differential misclassification. Such bias would most likely be toward the null.

Furthermore, it is unlikely that the self-reported multisite musculoskeletal pain or discomfort status would result in differential misclassification, as there is no reason to assume that participants would intentionally misreport their experiences of pain or discomfort. In line with the questionnaire wording, our outcome definition included both “pain” and “discomfort.” This approach captures a broader range of musculoskeletal symptoms that are still functionally relevant. While this may increase sensitivity by capturing a broader range of experiences, it also introduces greater variability in symptom severity and may complicate direct comparisons with studies focusing exclusively on “pain.” Pain was assessed over the past 12 months; however, data on pain duration were not available, which limits the distinction between acute and chronic pain. In our study, anatomical regions were grouped into composite categories (e.g., hips/knees/ankles counted as one site). This means that individuals with pain in several joints within the same region would still be classified as having one pain site. While this approach increases feasibility in large-scale surveys, it may underestimate the extent of localized pain burden and could influence the interpretation of single site versus multisite pain.

In addition, pain is fluctuating by nature, and different studies use different methodologies when gathering information about the experience of pain. Reporting musculoskeletal pain or discomfort within the last 12 months may be a bit imprecise and subject to recall bias. This could lead to nondifferential misclassification and a bias toward the null. Hanvold et al. measured pain status every 4 months during 20 follow-ups to make the measure more robust and found it was quite stable over time [5]. Despite our use of a less precise measure, the experience of pain in our study aligns with previous research findings [2, 3, 19].

Several of the nonsignificant estimates are likely due to Type II errors, particularly among males, given the relatively small sample size (*n* = 756), with only 26% reporting multisite pain compared to females (*n* = 1077), among whom 37% reported multisite musculoskeletal pain or discomfort. With an even larger study population, we might have obtained statistically significant ORs among males.

Two sensitivity analyses were performed on the outcome variable (see Tables S1 and S2 in the supporting information).  Table S1 presents an analysis in which the cutoff point for multisite musculoskeletal pain was changed from 2–4 pain sites to 3–4 pain sites. This adjustment resulted in a prevalence of multisite musculoskeletal pain of 13%. Overall, the results did not change notably; however, some differences emerged in the stratified analyses. The pattern observed in the sensitivity analysis for females was consistent with the original analysis, although the results were no longer statistically significant—likely due to the lower proportion of individuals classified as experiencing pain. Among males, the association between low physical activity and multisite pain was unclear. Interestingly, the sensitivity analysis suggests that low physical activity might act as a protective factor against experiencing pain. It is conceivable that individuals reporting pain in 3–4 anatomical sites represent a distinct clinical subgroup in which as-yet unidentified somatic and psychological factors contribute to the pain presentation—potentially explaining the observed inverse association.

Table S2 presents an analysis in which the cutoff level for pain categorization was modified. Participants were classified as experiencing no pain if they responded “none” or “very little” and as experiencing pain if they responded “a little bit,” “some,” “a great deal,” “much,” or “extremely much.” Overall, altering the pain cutoff did not substantially affect the results; however, as with the previous sensitivity analysis, the associations were no longer statistically significant.

Our study population consisted of 1833 participants, since it was a criterion that participants had answered questions regarding physical activity at least once at Age 15, 18, or 21 and pain status at Age 28. This resulted in a nonparticipation rate of 51%. The follow-up analysis revealed that in our study population, 59% were females and 7% had primary school as the highest educational level at Age 28, whereas in the source population, 48% were females and 13% had primary school as the highest educational level. As expected, some selection due to nonparticipation was present. The participants are more often females and have higher educational and income levels compared to nonparticipants [26, 36]. We do not think that this potential selection explains the association between physical inactivity and multisite musculoskeletal pain or discomfort; it will probably only lead to bias toward the null.

Some residual confounding may exist in this study due to missing data. Furthermore, the measurement of pain at baseline is insufficient, as data on low back pain and muscle pain in general were the only available information on pain at that age point. Additionally, stress and anxiety, known risk factors for multisite musculoskeletal pain [7, 9, 11, 13, 19], were not accounted for and may confound the relationship between physical activity and multisite musculoskeletal pain or discomfort.

Nevertheless, the findings of this follow-up study highlight the importance of remaining physically active during adolescence and young adulthood and maintaining these healthy habits into adulthood. Physical activity offers numerous health benefits, and with a small proportion of adolescents meeting WHO recommendations, there is significant potential for prevention [15]. Furthermore, people with multisite musculoskeletal pain tend to seek more health care, and an increased number of pain sites is associated with a higher number of long-term health care contacts [37]. Therefore, our findings of an association between physical inactivity and musculoskeletal pain or discomfort have implications for public health, health care costs, and clinical practices.

### 4.2. Conclusion

In this study population, 32% reported multisite musculoskeletal pain or discomfort within the last 12 months at age 28, and this was significantly associated with the level of physical activity during youth and young adulthood. Adolescents and young adults aged 15, 18, and 21 with a low level of physical activity were more likely to experience multisite musculoskeletal pain or discomfort at Age 28 compared to their more active peers. Those who remained physically inactive throughout adolescence and young adulthood had higher odds of experiencing multisite musculoskeletal pain or discomfort at Age 28 in comparison to those who maintained physical activity. Moreover, statistically significant associations were seen in females but not in males. These results have important implications for public health and health care costs, and with a small proportion of adolescents meeting WHO recommendations, there is a significant potential for prevention.

## Figures and Tables

**Table 1:** Descriptive statistics of the study population.

**Source population (*n* = 3681)**		**Total**	**Females**	**Males**

Study population		*n* = 1833	*n* = 1077 (59)	*n* = 756 (41)
Multisite musculoskeletal pain or discomfort	*n* = 1833			
Absent, *n* (%)		1239 (68)	682 (63)	557 (74)
Present, *n* (%)		594 (32)	395 (37)	199 (26)
Physical activity level				
15 years old	*n* = 1708			
None		28 (2)	15 (1)	13 (2)
1/2 h, *n* (%)		68 (4)	43 (4)	25 (4)
1 h, *n* (%)		187 (11)	116 (12)	71 (10)
2–3 h, *n* (%)		463 (27)	305 (30)	158 (23)
4–6 h, *n* (%)		578 (34)	348 (35)	230 (33)
7 h or more, *n* (%)		384 (22)	180 (18)	204 (29)
18 years old	*n* = 1476			
None		57 (4)	31 (3)	26 (4)
1/2 h, *n* (%)		79 (5)	47 (5)	32 (5)
1 h, *n* (%)		204 (14)	144 (16)	60 (10)
2–3 h, *n* (%)		458 (31)	302 (34)	156 (27)
4–6 h, *n* (%)		438 (30)	252 (28)	186 (32)
7 h or more, *n* (%)		240 (16)	117 (13)	123 (21)
21 years old	*n* = 1298			
None		76 (6)	40 (5)	36 (7)
1/2 h, *n* (%)		102 (8)	71 (9)	31 (6)
1 h, *n* (%)		168 (13)	112 (14)	56 (11)
2–3 h, *n* (%)		433 (33)	296 (37)	137 (28)
4–6 h, *n* (%)		340 (26)	208 (26)	132 (27)
7 h or more, *n* (%)		179 (14)	77 (10)	102 (21)
Accumulated physical activity (Ages 15, 18 and 21)	*n* = 1264			
Active maintainers, *n* (%)		251 (20)	124 (16)	127 (26)
Inactive maintainers, *n* (%)		267 (21)	192 (25)	75 (15)
Fluctuaters, *n* (%)		746 (59)	450 (59)	296 (59)
Depressive symptoms^a^, mean (SD)	*n* = 1823	2.3 (1.8)	2.6 (1.9)	2 (1.6)
Smoking (Ages 18 and/or 21)	*n* = 1833			
No, *n* (%)		1422 (78)	845 (78)	577 (76)
Yes, *n* (%)		411 (22)	232 (22)	179 (24)
Pain at baseline (Age 15)	*n* = 1697			
Absent, *n* (%)		1238 (73)	703 (70)	535 (77)
Present, *n* (%)		459 (27)	302 (30)	157 (23)
Socioeconomic status				
Parents' highest education (baseline)^b^	*n* = 1787			
< 10 years, *n* (%)		228 (13)	139 (13)	89 (12)
10–12 years, *n* (%)		829 (46)	508 (49)	321 (43)
13–15 years, *n* (%)		612 (34)	337 (32)	275 (37)
> 15 years, *n* (%)		118 (7)	64 (6)	54 (8)
Own education level as a 28-year-old^b^	*n* = 1830			
< 10 years, *n* (%)		120 (7)	59 (5)	61 (8)
10–12 years, *n* (%)		614 (33)	296 (28)	318 (42)
13–15 years, *n* (%)		774 (42)	520 (48)	254 (34)
> 15 years, *n* (%)		322 (18)	201 (19)	121 (16)
Equivalized childhood household income (at Ages 7–10), kr., mean (SD)	*n* = 1808	95,954 (38,137)	95,462 (33,153)	96,650 (44,237)

Abbreviation: SD, standard deviation.

^a^Average of 3 rounds, 15, 18, and 21 years old.

^b^< 10 years correspond to primary school, 10–12 years correspond to high school, technical school, or vocational education, 13–15 years correspond to short or medium-long further education (e.g., bachelor), and > 15 years correspond to long further education (e.g., master or PhD).

**Table 2:** Descriptive statistics of lost-to-follow-up.

	Source population		Study population	
Sex	*n* = 3681		*n* = 1833	
Females, *n* (%)		1777 (48)		1077 (59)
Males, *n* (%)		1904 (52)		756 (41)
Socioeconomic status				
Parents' highest education	*n* = 3507		*n* = 1787	
< 10 years, *n* (%)		520 (15)		228 (13)
Own education level as a 28-year-old	*n* = 3672		*n* = 1830	
< 10 years, *n* (%)		458 (13)		120 (7)
Equivalized childhood household income, mean (SD)	*n* = 3586	94,547 (37,496)	*n* = 1808	95,954 (38,136)

Abbreviation: SD, standard deviation.

**Table 3:** Associations between physical activity levels during adolescence and multisite musculoskeletal pain or discomfort at Age 28.

	Total		Females		Males	
	Crude^a^	Adjusted^b^	Crude^a^	Adjusted^b^	Crude^a^	Adjusted^b^
Physical activity level						
15 years old						
High	Ref.	Ref.	Ref.	Ref.	Ref.	Ref.
Low	1.5 (1.2–1.8)	1.4 (1.1–1.8)	1.4 (1.1–1.8)	1.3 (1.1–1.8)	1.4 (1.0–2.0)	1.3 (0.9–2.0)
Log-likelihood	−1064	−971	−655	−598	−401	−364
18 years old						
High	Ref.	Ref.	Ref.	Ref.	Ref.	Ref.
Low	1.5 (1.2–1.9)	1.3 (1.1–1.7)	1.6 (1.2–2.2)	1.5 (1.1–2.0)	1.2 (0.8–1.7)	1.0 (0.6–1.5)
Log-likelihood	−914	−796	−578	−508	−327	−280
21 years old						
High	Ref.	Ref.	Ref.	Ref.	Ref.	
Low	1.4 (1.1–1.8)	1.3 (1.1–1.8)	1.3 (0.9–1.7)	1.3 (0.9–1.8)	1.4 (0.9–2.1)	1.3 (0.8–2.0)
Log-likelihood	−805	−708	−520	−463	−278	−239
Accumulated physical activity (Ages 15, 18 and 21)						
Active maintainers	Ref.	Ref.	Ref.	Ref.	Ref.	Ref,
Fluctuaters	1.7 (1.2–2.4)	1.4 (1.0–2.1)	1.7 (1.1–2.6)	1.4 (0.9–2.3)	1.6 (0.9–3.7)	1.4 (0.8–3.2)
Inactive maintainers	2.3 (1.5–3.4)	1.9 (1.3–2.9)	2.2 (1.3–3.6)	1.9 (1.1–3.2)	1.9 (0.9–2.6)	1.6 (0.8–2.4)
Log-likelihood	−769	−715	−489	−463	−274	−246
Depressive symptoms	1.2 (1.1–1.2)		1.1 (1.1–1.2)		1.2 (1.1–1.3)	
Smoking (Age 18 and/or 21)						
No	Ref.		Ref.		Ref.	
Yes	1.6 (1.2–1.9)		1.7 (1.3–2.3)		1.4 (0.9–2.0)	
Pain at baseline (Age 15)						
Absent	Ref.		Ref.		Ref.	
Present	1.5 (1.2–1.9)		1.5 (1.2–2.0)		1.4 (0.9–2.0)	
Socioeconomic status						
Parents' highest education (baseline)						
< 10 years	Ref.		Ref.		Ref.	
10–12 years	0.7 (0.5–0.9)		0.8 (0.6–1.2)		0.5 (0.3–0.9)	
13–15 years	0.5 (0.4–0.8)		0.7 (0.4–1.1)		0.4 (0.2–0.7)	
> 15 years	0.6 (0.4–1.0)		0.6 (0.3–1.1)		0.7 (0.3–1.4)	
Own education level as a 28-year-old						
< 10 years	Ref.		Ref.		Ref.	
10–12 years	0.7 (0.4–1.0)		0.8 (0.4–1.3)		0.5 (0.3–0.9)	
13–15 years	0.5 (0.3–0.7)		0.5 (0.3–0.8)		0.4 (0.2–0.8)	
> 15 years	0.5 (0.3–0.7)		0.4 (0.2–0.8)		0.4 (0.2–0.8)	
Equivalized childhood household income (kr.)	1.0 (1.0–1.0)		1.0 (1.0–1.1)		1.0 (1.0–1.1)	

*Note:* Crude and adjusted estimates represent odds ratios and 95% confidence intervals.

^a^Crude estimates for all included variables.

^b^Estimates adjusted for depressive symptoms, smoking, pain at baseline, and socioeconomic status.

## Data Availability

The data that support the findings of this study are available on request from the corresponding author. The data are not publicly available due to privacy or ethical restrictions.

## Supporting Information

The supporting information includes the STROBE checklist and two tables that show sensitivity analyses of the outcome variable.

A sensitivity analysis, see table S1 in supporting information, of the outcome variable was performed by changing the cutoff point for multisite musculoskeletal pain from 2–4 pain sites to 3–4 pain sites. It provides further evidence for the findings and supports the conclusion, since the results did not alter notably when changing the cutoff point for pain.

Another sensitivity analysis, see table S2 in supporting information, of the outcome variable was conducted by altering the cutoff level for pain categorization. Participants were classified as experiencing no pain if they responded with “none” or “very little” and as experiencing pain if they responded with “a little bit,” “some,” “a great deal,” “much,” or “extremely much.”

## References

[1] Carnes D., Parsons S., Ashby D. (2007). Chronic Musculoskeletal Pain Rarely Presents in a Single Body Site: Results From a UK Population Study. *Rheumatology*.

[2] Bazett-Jones D. M., Rathleff M. S., Holden S. (2019). Associations Between Number of Pain Sites and Sleep, Sports Participation, and Quality of Life: A Cross-Sectional Survey of 1021 Youth From the Midwestern United States. *BMC Pediatrics*.

[3] Eckhoff C., Straume B., Kvernmo S. (2017). Multisite Musculoskeletal Pain in Adolescence and Later Mental Health Disorders: A Population-Based Registry Study of Norwegian Youth: The NAAHS Cohort Study. *BMJ Open*.

[4] Hoftun G. B., Romundstad P. R., Zwart J. A., Rygg M. (2011). Chronic Idiopathic Pain in Adolescence--High Prevalence and Disability: The Young HUNT Study 2008. *Pain*.

[5] Hanvold T. N., Lunde L. K., Koch M., Wærsted M., Veiersted K. B. (2016). Multisite Musculoskeletal Pain Among Young Technical School Students Entering Working Life. *BMC Musculoskeletal Disorders*.

[6] Kamaleri Y., Natvig B., Ihlebaek C. M., Benth J. S., Bruusgaard D. (2009). Change in the Number of Musculoskeletal Pain Sites: A 14-Year Prospective Study. *Pain*.

[7] Malmborg J. S., Bremander A., Olsson M. C., Bergman A. C., Brorsson A. S., Bergman S. (2019). Worse Health Status, Sleeping Problems, and Anxiety in 16-Year-Old Students Are Associated With Chronic Musculoskeletal Pain at Three-Year Follow-Up. *BMC Public Health*.

[8] Holley A. L., Law E. F., Tham S. W. (2013). Current Smoking as a Predictor of Chronic Musculoskeletal Pain in Young Adult Twins. *The Journal of Pain*.

[9] Huguet A., Tougas M. E., Hayden J., McGrath P. J., Stinson J. N., Chambers C. T. (2016). Systematic Review With Meta-Analysis of Childhood and Adolescent Risk and Prognostic Factors for Musculoskeletal Pain. *Pain*.

[10] Puroila A., Paananen M., Taimela S., Järvelin M. R., Karppinen J. (2015). Lifestyle-Factors in Adolescence as Predictors of Number of Musculoskeletal Pain Sites in Adulthood: A 17-year Follow-Up Study of a Birth Cohort. *Pain Medicine*.

[11] Andreucci A., Campbell P., Dunn K. M. (2021). Are Psychological Symptoms a Risk Factor for Musculoskeletal Pain in Adolescents?. *European Journal of Pediatrics*.

[12] Hoftun G. B., Romundstad P. R., Rygg M. (2012). Factors Associated With Adolescent Chronic Non-Specific Pain, Chronic Multisite Pain, and Chronic Pain With High Disability: The Young-HUNT Study 2008. *The Journal of Pain*.

[13] Jussila L., Paananen M., Näyhä S. (2014). Psychosocial and Lifestyle Correlates of Musculoskeletal Pain Patterns in Adolescence: A 2-Year Follow-Up Study. *European Journal of Pain*.

[14] Bull F. C., Al-Ansari S. S., Biddle S. (2020). World Health Organization 2020 Guidelines on Physical Activity and Sedentary Behaviour. *British Journal of Sports Medicine*.

[15] Guthold R., Stevens G. A., Riley L. M., Bull F. C. (2020). Global Trends in Insufficient Physical Activity Among Adolescents: A Pooled Analysis of 298 Population-Based Surveys With 1·6 Million Participants. *The Lancet Child & Adolescent Health*.

[16] Corder K., Winpenny E., Love R., Brown H. E., White M., Sluijs E. V. (2019). Change in Physical Activity From Adolescence to Early Adulthood: A Systematic Review and Meta-Analysis of Longitudinal Cohort Studies. *British Journal of Sports Medicine*.

[17] Ortega F. B., Konstabel K., Pasquali E. (2013). Objectively Measured Physical Activity and Sedentary Time During Childhood, Adolescence and Young Adulthood: A Cohort Study. *PLoS One*.

[18] Paananen M. V., Auvinen J. P., Taimela S. P. (2010). Psychosocial, Mechanical, and Metabolic Factors in Adolescents’ Musculoskeletal Pain in Multiple Locations: A Cross-Sectional Study. *European Journal of Pain*.

[19] Paananen M. V., Taimela S. P., Auvinen J. P. (2010). Risk Factors for Persistence of Multiple Musculoskeletal Pains in Adolescence: A 2-Year Follow-Up Study. *European Journal of Pain*.

[20] Ahlholm V.-H., Rönkkö V., Ala-Mursula L., Karppinen J., Oura P. (2021). Modeling the Multidimensional Predictors of Multisite Musculoskeletal Pain Across Adulthood—A Generalized Estimating Equations Approach. *Frontiers in Public Health*.

[21] Guddal M. H., Stensland S., Småstuen M. C., Johnsen M. B., Zwart J. A., Storheim K. (2017). Physical Activity Level and Sport Participation in Relation to Musculoskeletal Pain in a Population-Based Study of Adolescents: The Young-HUNT Study. *Orthopaedic Journal of Sports Medicine*.

[22] Smedbråten K., Grotle M., Jahre H. (2022). Lifestyle Behaviour in Adolescence and Musculoskeletal Pain 11 Years Later: The Trøndelag Health Study. *European Journal of Pain*.

[23] Wedderkopp N., Kjaer P., Hestbaek L., Korsholm L., Leboeuf-Yde C. (2009). High-Level Physical Activity in Childhood Seems to Protect Against Low Back Pain in Early Adolescence. *The Spine Journal*.

[24] Gallagher K. M., Howie E. K., Carney M. (2023). Musculoskeletal Pain Latent Classes and Biopsychosocial Characteristics Among Emerging Adults. *BMC Musculoskeletal Disorders*.

[25] Heikkala E., Paananen M., Taimela S., Auvinen J., Karppinen J. (2019). Associations of Co-Occurring Psychosocial and Lifestyle Factors With Multisite Musculoskeletal Pain During Late Adolescence-A Birth Cohort Study. *European Journal of Pain*.

[26] Winding T. N., Andersen J. H., Labriola M., Nohr E. A. (2014). Initial Non-Participation and Loss to Follow-Up in a Danish Youth Cohort: Implications for Relative Risk Estimates. *Journal of Epidemiology & Community Health*.

[27] Pedersen C. B. (2011). The Danish Civil Registration System. *Scandinavian Journal of Public Health*.

[28] Fendrich M., Weissman M. M., Warner V. (1990). Screening for Depressive Disorder in Children and Adolescents: Validating the Center for Epidemiologic Studies Depression Scale for Children. *American Journal of Epidemiology*.

[29] Derogatis L. R. (1983). SCL-90-R: Administration, Scoring and Procedures. *Manual II for the R (Revised) Version and Other Instruments of the Psychopathology Rating Scale Series*.

[30] Kempel M. K., Winding T. N., Böttcher M., Andersen J. H. (2022). Evaluating the Association Between Socioeconomic Position and Cardiometabolic Risk Markers in Young Adulthood by Different Life Course Models. *BMC Public Health*.

[31] Veldman K., Reijneveld S. A., Hviid Andersen J. (2022). The Timing and Duration of Depressive Symptoms From Adolescence to Young Adulthood and Young Adults’ NEET Status: The Role of Educational Attainment. *Social Psychiatry and Psychiatric Epidemiology*.

[32] Winding T. N., Bøjstrup J., Christensen S. (2025). Adolescent Friendships and Their Impact on Self-Rated Health in Early Adulthood. A Prospective Cohort Study. *Scandinavian Journal of Public Health*.

[33] Kamaleri Y., Natvig B., Ihlebaek C. M., Benth J. S., Bruusgaard D. (2008). Number of Pain Sites Is Associated With Demographic, Lifestyle, and Health-Related Factors in the General Population. *European Journal of Pain*.

[34] Kamada M., Abe T., Kitayuguchi J. (2016). Dose-Response Relationship Between Sports Activity and Musculoskeletal Pain in Adolescents. *Pain*.

[35] Gatchel R. J., Peng Y. B., Peters M. L., Fuchs P. N., Turk D. C. (2007). The Biopsychosocial Approach to Chronic Pain: Scientific Advances and Future Directions. *Psychological Bulletin*.

[36] Goldberg M., Chastang J. F., Leclerc A. (2001). Socioeconomic, Demographic, Occupational, and Health Factors Associated With Participation in a Long-Term Epidemiologic Survey: A Prospective Study of the French GAZEL Cohort and Its Target Population. *American Journal of Epidemiology*.

[37] Mose S., Kent P., Smith A., Andersen J. H., Christiansen D. H. (2021). Number of Musculoskeletal Pain Sites Leads to Increased Long-Term Healthcare Contacts and Healthcare Related Costs-A Danish Population-Based Cohort Study. *BMC Health Services Research*.

