# Supplementary material for: How Does Physical Activity During Youth Affect the Development of Multisite Musculoskeletal Pain or Discomfort in Young Adults?
Source: Pain Res Manag. 2025 Nov 29;2025:8810256. doi: 10.1155/prm/8810256 (PMC12681414; doi:10.1155/prm/8810256)
Supplement: Supporting Information — Additional supporting information can be found online in the Supporting Information section. [file 8810256.f1.zip › supplemantary materials.docx]

| **Table S1: Sensitivity analysis of the outcome variable by changing the cut-off point for multisite musculoskeletal pain** | | | | |  |  |  |  |
| --- | --- | --- | --- | --- | --- | --- | --- | --- |
|  |  |  |  |  |  |  |  |  |
|  | | | **Total** |  | **Females** |  | **Males** |  |
|  | | | Crude | Adjusted | Crude | Adjusted | Crude | Adjusted |
|  | | | *OR (95 % CI)* | *OR (95 % CI)* | *OR (95 % CI)* | *OR (95 % CI)* | *OR (95 % CI)* | *OR (95 % CI)* |
| **Physical activity level** | | |  |  |  |  |  |  |
| 15 years old | | |  |  |  |  |  |  |
| High | | | Ref. | Ref. | Ref. | Ref. | Ref. | Ref. |
| Low, | | | 1.02 (0.8-1.4) | 0.86 (0.6-1.2) | 1.0 (0.7-1.4) | 0.9 (0.6-1.3) | 0.9 (0.5-1.5) | 0.6 (0.3-1.2) |
| 18 years old | | |  |  |  |  |  |  |
| High | | | Ref. | Ref. | Ref. | Ref. | Ref. | Ref. |
| Low | | | 1.5 (1.1-2.0) | 1.2 (0.8-1.7) | 1.5 (1.0-2.3) | 1.4 (0.9-2.1) | 1.1 (0.6-1.9) | 0.8 (0.4-1.5) |
| 21 years old | | |  |  |  |  |  |  |
| High | | | Ref. | Ref. | Ref. | Ref. | Ref. |  |
| Low | | | 1.3 (0.9-1.8) | 1.2 (0.8-1.8) | 1.2 (0.8-1.9) | 1.3 (0.8-2.2) | 1.1 (0.5-2.0) | 0.7 (0.1-1.6) |
|  | | |  |  |  |  |  |  |
| **Accumulated physical activity** | | |  |  |  |  |  |  |
| Active maintainers | | | Ref. | Ref. | Ref. | Ref. | Ref. | Ref, |
| Fluctuaters | | | 1.9 (1.1-3.0) | 1.6 (0.9-2.7) | 2.1 (1.4-4.2) | 1.8 (0.9-3.8) | 1.4 (0.6-2.9) | 1.2 (0.5-2.9) |
| Inactive maintainers | | | 1.7 (0.9-3.1) | 1.4 (0.7-2.6) | 2.1 (0.9-4.5) | 1.8 (0.8-4.0) | 0.5 (0.1-2.0) | 0.4 (0.1-1.6) |

Multisite musculoskeletal pain defined as 3-4 pain sites, n = 236 (13 %) of the study population

A sensitivity analysis of the outcome variable was performed by changing the cut-off point for multisite musculoskeletal pain from 2-4 pain sites to 3-4 pain sites.

**Table S2: Sensitivity analysis of the outcome variable by changing the cut off level for pain**

|  | **Total** |  | **Females** |  | **Males** |  |
| --- | --- | --- | --- | --- | --- | --- |
|  | Crude | Adjusted | Crude | Adjusted | Crude | Adjusted |
|  | *OR (95 % CI)* | *OR (95 % CI)* | *OR (95 % CI)* | *OR (95 % CI)* | *OR (95 % CI)* | *OR (95 % CI)* |
| **Physical activity level** |  |  |  |  |  |  |
| 15 years old |  |  |  |  |  |  |
| High | Ref. | Ref. | Ref. | Ref. | Ref. | Ref. |
| Low, | 1.2 (1.01-1.5) | 1.2 (0.9-1.4) | 1.1 (0.9-1.4) | 1.04 (0.8-1.3) | 1.3 (0.9-1.8) | 1.3 (0.9-1.8) |
| 18 years old |  |  |  |  |  |  |
| High | Ref. | Ref. | Ref. | Ref. | Ref. | Ref. |
| Low | 1.3 (1.05-1.6) | 1.2 (0.9-1.5) | 1.3 (1.02-1.8) | 1.3 (0.8-1.7) | 1.1 (0.8-1.5) | 0.9 (0.7-1.4) |
| 21 years old |  |  |  |  |  |  |
| High | Ref. | Ref. | Ref. | Ref. | Ref. |  |
| Low | 1.3 (0.95-1.49) | 1.1 (0.9-1.5) | 1.08 (0.8-1.4) | 1.03 (0.8-1.4) | 1.3 (0.9-1.8) | 1.2 (0.8-1.8) |
|  |  |  |  |  |  |  |
| **Accumulated physical activity** |  |  |  |  |  |  |
| Active maintainers | Ref. | Ref. | Ref. | Ref. | Ref. | Ref, |
| Fluctuaters | 1.3 (0.88-1.57) | 1.07 (0.8-1.4) | 0.9 (0.8-1.9) | 0.9 (0.7-1.8) | 1.3 (0.9-2.02) | 1.2 (0.8-1.9) |
| Inactive maintainers | 1.3 (0.9-1.9) | 1.2 (0.8-1.7) | 1.2 (0.8-1.9) | 1.1 (0.7-1.8) | 1.2 (0.7-2.1) | 1.07 (0.6-2.0) |

A sensitivity analysis of the outcome variable was conducted by altering the cut-off level for pain categorization. Participants were classified as experiencing no pain if they responded with “none” or “very little,” and as experiencing pain if they responded with “a little bit,” “some,” “a great deal,” “much,” or “extremely much.”
